# Supplementary material for: Construction of Cisplatin-18-Crown-6 Complexes Through Supramolecular Chemistry to Improve Solubility, Stability, and Antitumor Activity
Source: Int J Mol Sci. 2024 Dec 14;25(24):13411. doi: 10.3390/ijms252413411 (PMC11678135; doi:10.3390/ijms252413411)
Supplement: Supplementary file 1 [file ijms-25-13411-s001.zip › ijms-3349822-supplementary.pdf]

Construction of cisplatin-18-crown-6 complexes through supramolecular chemistry to improve solubility, stability, and antitumor activity

Yue Gao, Yeqi Huang, Chuanyu Ren, Si Xiong, Xia Guo, Ziyu Zhao*, Ling Guo, Zhengwei Huang*

1. Standard curve of DDP

Precise quantities of DDP powder were weighed and dissolved in 30% PEG400 aqueous solution to prepare DDP solutions at varying concentrations (0.4, 0.8, 1.2, 1.6, 2.0 mg/ml). UV absorption was measured at 301 nm to record absorbance, and a standard curve of DDP concentration versus absorbance was constructed.

2. Standard curve of DDP

The UV absorbance of DDP at 301 nm was measured across a gradient of concentrations to establish a calibration curve. As depicted in Figure S1, linear regression analysis yielded the standard curve equation $Y = 0.3459X+0.0827$ (R^2>0.99), demonstrating an excellent fit. This standard curve was valid within a concentration range of 0.4 to 2 mg/ml and was subsequently utilized for quantifying DDP levels in further experiments.

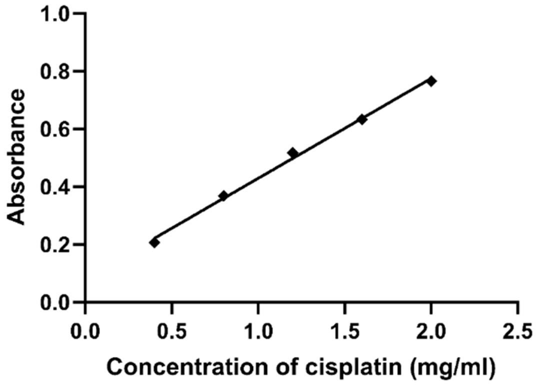

Figure S1. Standard curve of cisplatin.

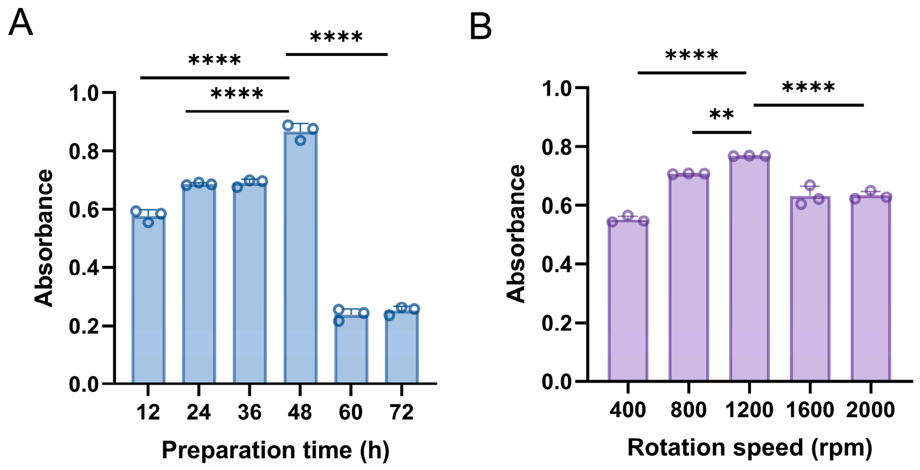

Figure S2. The optimization of preparation time (A) and rotation rate (B) of DDP@18-crown-6 complex by UV absorbance at 301 nm (n = 3). ANOVA or t tests were utilized to determine whether there are significant differences between the data. P value style: **$p < 0.01$; ****$p < 0.0001$.
